# REAL-WORLD REPRESENTATIVENESS OF CANADIAN RESEARCH SUBJECTS WITH MILD COGNITIVE IMPAIRMENT

**DOI:** 10.1093/geroni/igz038.2446

**Published:** 2019-11-08

**Authors:** Vivian Huang, David B Hogan, Zahinoor Ismail, Colleen J Maxwell, Eric E Smith, Brandy L Callahan

**Affiliations:** 1 Ryerson University, Toronto, Ontario, Canada; 2 University of Calgary, Calgary, Alberta, Canada; 3 University of Waterloo, Waterloo, Ontario, Canada

## Abstract

Studies of mild cognitive impairment (MCI) utilize stringent inclusion/exclusion criteria which may impact the generalizability of findings to the broader clinical population. We compared characteristics of MCI patients in a Canadian memory clinic in Calgary to MCI research participants in published Canadian studies to assess the representativeness of research samples. Clinic participants included 555 MCI patients from the Prospective Study for Persons with Memory Symptoms registry. Research participants included 4,981 individuals with MCI retained from a systematic literature review of 112 peer-reviewed empirical Canadian studies. Clinic patients and research participants were diagnosed with MCI using similar diagnostic criteria (i.e., from the NIA-AA, or Petersen criteria). Both samples were compared on baseline demographic variables, medical and psychiatric comorbidities, and global cognitive performance using chi-square tests and t-tests with weighted means. Diverse presumed causes were noted among clinic patients. Clinic patients were younger, more likely to be male, and more educated than research participants (ds: 0.22-0.98). Psychiatric disorders, traumatic brain injury, and sensory impairments were common in clinic patients (up to 83%), but participants with these conditions were excluded from approximately 80% of studies in the systematic review. Clinic patients performed significantly worse on two global cognitive assessments (ds: 0.53 – 1.27). Stringent eligibility criteria such as used in Canadian MCI research studies would exclude a considerable subset of MCI patients seen in our referral clinic. This may have contributed to the disparities between the clinical and research cohorts in the cognitive measures examined. The implications of these findings will be discussed.

